# The Tango of Problem Formulation: A Patient’s/Researcher’s Reflection on an Action Design Research Journey

**DOI:** 10.2196/16916

**Published:** 2020-07-14

**Authors:** Michael B Twomey, David Sammon, Tadhg Nagle

**Affiliations:** 1 Business Information Systems Cork University Business School University College Cork Cork Ireland

**Keywords:** action design research, patient, reflection, problem formulation, checklist, cystic fibrosis

## Abstract

This paper reports on the reflection of the lead researcher, a 48-year-old patient with cystic fibrosis (CF), and aims to portray his real-life experience of a 10-month action design research (ADR) project. Playing a dual role, as both a patient and researcher, the lead researcher reflects deeply on his ADR experience with particular emphasis on the problem formulation stage of creating a simple yet impactful checklist to aid memory recall of CF patients or caregivers during a medical appointment. Using Driscoll’s model of reflection, a real-life unsanitized ADR experience is carefully imparted via a series of 4 vignettes, including 4 key learnings, which highlight the connection between a meticulous considered approach to problem formulation and truly effective outcomes. By providing this rich account of problem formulation within ADR, it is hoped that this reflection will help researchers to better understand the complexity of problem formulation in design-oriented research; to avoid making assumptions and becoming fixated on solutions; and to move instead to an end point where several possible ways of examining a problem have been considered, explored, and understood—an end point where successful end results are reached through grit and determination. This paper advocates for the inclusion and portrayal of the actual realities or ups and downs of this dynamic and evolving stage of ADR, capturing the often-tacit knowledge of problem formulation and begetting a sense of realism and humanity to ADR serving as knowledge contributions in their own right. The lead researcher is the patient and researcher in this ADR project. This is my story!

## Introduction

It is a windy Saturday afternoon in February 2020, and I am busy packing up a box of checklist booklets that I designed to aid the memory recall or information retrieval of patients with cystic fibrosis (CF) and their caregivers during their medical appointments. The box is on its way to the Royal London Children’s Hospital, Whitechapel, London, for distribution to caregivers of patients with CF. Caregivers know the reality of having a sick child in a medical appointment, the stress of trying to remember medical history, and the difficulty of trying to recall afterward what took place within a medical appointment. I tape up the box carefully, making sure it is secure for its journey ahead.

A journey that would not have come to pass had I not been invited to present my action design research (ADR) study at the International CF Clinical Conference held in Killarney, Ireland, on January 30, 2020. Out of that, came the dispatching of the checklist booklet to Spain, Sweden, Israel, and Australia. There is no doubt that the checklist booklet is beginning to travel far and wide. Earlier, in January 2019, the booklet was distributed by Cystic Fibrosis Ireland to every patient or caregiver within the Republic of Ireland (1300 patients with CF). Afterward, in April 2019, it was shipped to the Czech Cystic Fibrosis Association and Austria for review.

Moreover, in October 2019, I was invited to present my research on the booklet to over 100 clinicians at a *Hot Topics in CF* conference held in Birmingham, England. Nevertheless, the real impact of the checklist booklet is best depicted by the mother of a 7-year-old child with CF:

We just wanted to say we received our medical appointment check list today, and we just wanted to say THANK YOU so much, we love it and it’s going to be incredibly handy for us, although it’s just a book to our little boy now, in a few years he’ll know how great and simple it is as well.

As I contemplate the same, a warm feeling envelops me, I feel I am beginning to make a difference, a difference to people like me.

The box is ready to go, a thought enters my mind, *So was my ADR journey easy*, the answer is definitely no. *Did it take determination and patience?* Yes, for sure! *And did I sometimes think of giving up? Did it test me to my limits?* I would have to say yes on both accounts. So how do I account for the success of the checklist booklet? Earlier I mentioned determination; I was unyielding in my quest to understand the problem, the problem that I and others like me experience every time we are in a medical appointment. To help you understand, however, I need to take you on a voyage, a voyage of reflection. It is only by coming on this journey that you will come to comprehend the end point, where through grit and determination, success can be achieved.

“Humans have always reflected on experiences and feelings” [1]. According to Boud et al [2] reflection is “an important human activity in which people recapture their experience, think about it, mull it over and evaluate it.” As a researcher, I felt an obligation to share my unsanitized lived experience of *problem formulation* in ADR, not only as a practitioner but also through the eyes of a patient researcher living with a chronic illness.

To this end, I found that a reflection would be the most appropriate instrument to aid me with the *mental process of trying to structure or restructure* [3] my real-life experience of *problem formulation*, to put together or capture the *existing knowledge or insights* [3] from the project that I lived through over a 10-month period. I hope that the insights imparted herein may serve not only as insightful to ADR practitioners in their *problem formulation* endeavors but also to highlight the importance of this stage of ADR in achieving successful outcomes. Moreover, I hope that any patients reading my reflection may be inspired to enter the stimulating world of research, making real-world impacts within their own patient communities, as I have strived to do in mine.

The paper is structured as follows. First, I present a very brief background on ADR, the methodology that I used in my research exploration, going somewhat deeper on the *problem formulation* stage of the methodology (the focus of my reflection). Next, I endeavor to set the scene, giving the reader a deep candid sense of the patient researcher behind the reflection, followed by a very brief section on why a reflection was the correct tool for my deliberations, and the rationale behind the model of reflection I selected. I subsequently organize my reflection through a series of 4 vignettes, which are used to explain the lessons that I learned from my experience of *problem formulation* within ADR and how crucial this was to the effects my research is having now. Finally, I bring my musings to a close in the concluding remarks section of the paper.

## Background

### Action Design Research

Design science research (DSR) accentuates a *construction-oriented* interpretation of information systems (IS) research, which at its core lies the design and build of innovative information technology (IT) artifacts and which is deemed appropriate when research aims to produce artifacts that address so called *wicked problems* or ill-structured problems [4]. Although this approach provides IS researchers with the ability to go beyond mere elucidation, toward research that spawns design knowledge relevant to practitioners [4], it still fails to “fully recognize the role of organizational context in shaping the design as well as shaping the deployed artefact” [5].

**Figure 1:**
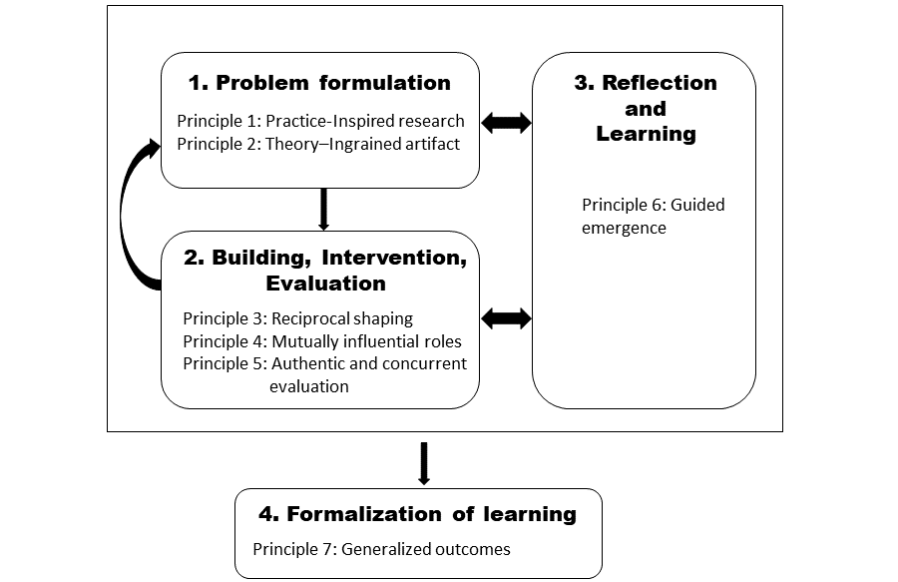
Action design research method: stages and principles.

In their seminal paper, Sein et al [5] proposed a variant of DSR (Figure 1), which is called action design research (ADR), and clearly acknowledged IT artifacts as “shaped by the interests, values, and assumptions of a wide variety” [6] of stakeholders at the same time retaining the essence of design research. ADR targets the creation of innovative artifacts in an *organizational context* but at the same time produces knowledge contributions from the intervention while tackling a problematic situation [4,7]. Sein and Rossi [8] argue that the “embedding of the context in the design through intervention in an organisation, a single-entry point (problem-centered), and, inductive epistemology, is the characteristics of ADR that validate knowledge claims of emergent knowledge co-produced with practice.”

ADR differs from other design approaches in so far as it draws on DSR, which centers on the utility of an artifact, and action research, which primarily focuses on learning from an environment, believing that “[t]he only way to produce conditions of practice is to move to practice” [9]. Moreover, at the core of ADR is an inquiry with rigorous evaluation, which is highly iterative in nature, consisting of nested loops [8], where each iteration concludes with a consideration of the artifact. This evaluation acts as the impetus for thorough *reflection and learning*, which then feeds back into *problem formulation*, thereby challenging *“*organizational participants’ existing ideas and assumptions about the artefact’s specific use context in order to create and improve the design*”* [5]. It is these very characteristics that make ADR so successful as a methodology.

It comes as no surprise, therefore, that ADR has been used very effectively in a wide array of research projects, and “because of its ever-expanding applications, the ADR concepts and process model continue to grow and evolve to meet the demands of new and challenging environments” [10], including those within the health domain. For example, the successful solution by Bretschneider et al [11] helps to leverage the innovative idea potential of patients better than traditional communication forums.

As a pragmatist who is “more interested in utility and usefulness than in an abstract notion of truth” [12], I deemed ADR as a suitable methodology for my research endeavors as it is grounded in and grounds research from practice, academia, and empirical data. Its primary focus is on learning from designing an artifact or intervention within an environment. This is important as it was imperative that the solution that I created worked within the complex environment of the medical appointment. My reflections later on in this paper reveal my experience of ADR as a patient and researcher and serve to contribute to the recent open and engaging discussions regarding problem *formulation* in ADR, consistent with the original authors’ contention, who contend that ADR “is still an open endeavor” [13]. First, let us delve into the *problem formulation* stage of ADR in greater depth.

### Problem Formulation

In 1998, Berthon et al [14] stated that *problem formulation* was the least researched of problem-solving activities. In 2018, Mullarkey and Hevner [10] reported on the challenges they had regarding the *problem formulation* stage of ADR. More specifically, they discovered that they “needed to better understand the problem space” [10] and maintained that the ADR model by Sein et al [5] required an explicit *diagnosis* stage (with a clear separation from design) model “to analyse the importance of the problem domain and the relevance of the IT solution class to research and practice” [10].

#### What Is Problem Formulation?

Simply put, *problem formulation* is the sum of problem ID (perception), problem definition (conceptualization), and problem structuring (instrumental reasoning). “The first requirement with any complex problem is to try and understand it as a totality. How has it arisen, and why? Where is it going and what route is it taking? Is it changing its nature or structure as it develops?” [15]. The *problem formulation* stage in ADR (Textbox 1 presents the tasks in the stage) identifies and conceptualizes (using both divergent and convergent metacognitive processes) a research opportunity based on existing theories and technologies [4], where the research activity is said to be problem inspired [16,17].

How important is the *problem formulation* stage of ADR? The value of a suitable definition has been established empirically [18]: “The more of the context of a problem that a scientist can comprehend, the greater are his chances of finding a truly adequate solution” [19]. Mintzberg et al [20] argue that *problem formulation* is “probably the single most important routine, since it determines in large part, however implicitly, the subsequent course of action.” However, Mitroff et al [21] maintained that problem forming and defining are as critical, if not more so than, problem solving. This is probably not surprising as our understanding of a problem greatly influences our selection of solutions [22] and helps avoid type III errors, solving the wrong problem [23].

**Tasks in the problem formulation stage of action design research [5].**

- Identify and conceptualize the research opportunity
- Formulate initial research questions
- Cast the problem as an instance of a class of problems
- Identify contributing theoretical bases and prior technology advances
- Secure long-term organizational commitment
- Set up roles and responsibilities

### Why Is Problem Formulation So Challenging?

According to Mitroff and Featheringham [24], one of the most important challenges of the problem-solving activity is solving the *wrong* problem by adapting a formulation that is either too narrow or inappropriate. So, one may well ask why *problem formulation* is so challenging? In Textbox 2, I have tried to encapsulate some of the key challenges to *problem formulation* that have been reported in literature.

### How Might Problem Formulation Be Done Better?

In their ADR process model, Mullarkey and Hevner [10] argued that every iteration should go through *problem formulation* and that reflection and learning should also be executed in every cycle—mainly as it informs the *problem formulation* for the next ADR iteration. They also included a fourth ADR stage *evolution*, as a means to address the very temporal and evolving nature of both the artifact and the problem environment.

Sein and Rossi [8] agreed that these modifications were a *valuable* addition to their initial model (which did not go into details) in terms of making it more *transparent* and *accessible to researchers;* they do however point out that this was intentional “because in launching a new method of doing DSR, we wanted to keep it at a broad enough level of abstraction to allow the actual application of the method to emerge in use.”.

**The challenges of problem formulation.**

- Problems, by their very nature, are complex and ill-structured [25-27].
- “In real life there is not a single, static, well-defined problem, but a constantly changing problem.” [28]
- “The capacity of the human mind for formulating and solving complex problems is very small compared with the size of the problems whose solution is required.” [29]
- Stakeholders may have conflicting interpretations of a problem resulting from different life experiences, competencies, goals, and values [23].
- Human biases: “fixated on these unwarranted assumptions, and this fixation interferes with the insight needed to solve the problem” [30].
- People often are too quick to move on to evaluative stages of problem solving rather than gaining a complete understanding of the problem [31].
- “We may be too ready to re-use features of known existing designs, rather than to explore the problem and generate new design features.” [32]
- People often only identify the most obvious symptoms, or those to which they are most sensitive, resulting in the problem being described inappropriately [24,27].
- Most companies are not adequately thorough in actually defining the problems they are trying to solve [33].
- “Problem formulation” has been shown to be highly dependent on the mode of problem presentation [34-36].
- Changing problem presentation modes has considerable effects on mental model formation [34], where Simon and Hayes found that “innocent changes in language had major effects on problem formulation.” [37]

When several stakeholders are affected by a problem, all viewpoints must be taken into consideration for a solution to be deemed successful [23]. After declaring the problematic gap, it is advisable to specify evidence supporting the presence of that gap. Indeed, Brody [38] raises the question, should problem statements include an “as evidenced by” clause? Moreover, Mitroff et al [21] advocated the use of assumptional analysis to question any assumptions, projections, and explanations lying beneath the problematic statement, whereas Lyles and Mitroff [25] also proposed that alternative views of the problem be sought to improve *problem formulation*.

The use of conceptual processing and mental models is also encouraged in DSR literature [39,40] to assist the *problem formulation* stage. Interestingly, Lesgold [41] discovered that experts expended additional time deciding an appropriate mental model of a problem than did novices. This may be explained because “what we understand and how we understand a situation depends on the information we bring to a given situation, and the longer we think about the situation the more its cognitive representation changes. It may be assumed that cognitive elaboration activates more schemata” [42] and hence enriched *problem formulation.*

Like any good story, it is important to first set the scene, facilitating an appreciation for key contextual elements of the story that I feel are important to comprehend, and so, we now move to the next section, that is, the research setting.

## Research Setting

It is 11:27 AM, it is a pleasant day outside, the sun is shining, summer shows visible signs of its arrival in the garden outside, with many perfumes and aromas creating an exciting olfactory feast. It seems appropriate that I start this paper on this date, May 25, 2019, as it is my sister Jane’s 50th birthday, and she was one of the key motivators and sources of inspiration for my research journey. Unfortunately, I cannot celebrate this special occasion with her as she passed away with CF on July 29, 1997. I feel an intense sense of sadness mixed with emotions of happiness as the memories of her sail across the horizon of my memory.

I have witnessed the effect that her passing has had on my family. I have seen the agony and the physical effects that the death of my sister has had on my dear parents. I will never forget that day. I will never forget the life-support machine flat-lining, the tears, the pain in that intensive care unit; it will live with me forever. The experience of her end has left an indelible mark on me not only because of the pain of her passing but also as I have CF myself. I have also lived through the hardship that one endures with CF. I have faced the dark shadows that come into a room when gasping for breath, where one’s mortality becomes all too real.

After my sister’s death, I had a choice; to let this disease define me and become a bitter, negative person who craved self-pity or to embrace the positive aspects that the disease had carved out or sculpted into my heart and mind. You might ask what do I mean by positive? I mean the appreciation for life, for family, for fun, and for being able to breathe. I mean the ability to empathize with others and to be compassionate to another person’s suffering. I wanted to make a difference, to give something back, as others have given to me, which has resulted in my own good health. I wanted to help others with CF who are going on their own difficult journey, a passage that has many dark and difficult days.

Although I admit that living with CF is not always easy, I have always been a fighter, I had to be! I would not give into myself or my condition, I love life too much. Life has so much to offer, alas many take their lives for granted. Moreover, I think when you are faced with the very serious question of your mortality at a young age, you learn to be truly grateful for the gift of life. That is the real reward of a chronic condition, and it became the match that ignited the fight and passions within me.

**Figure 2:**
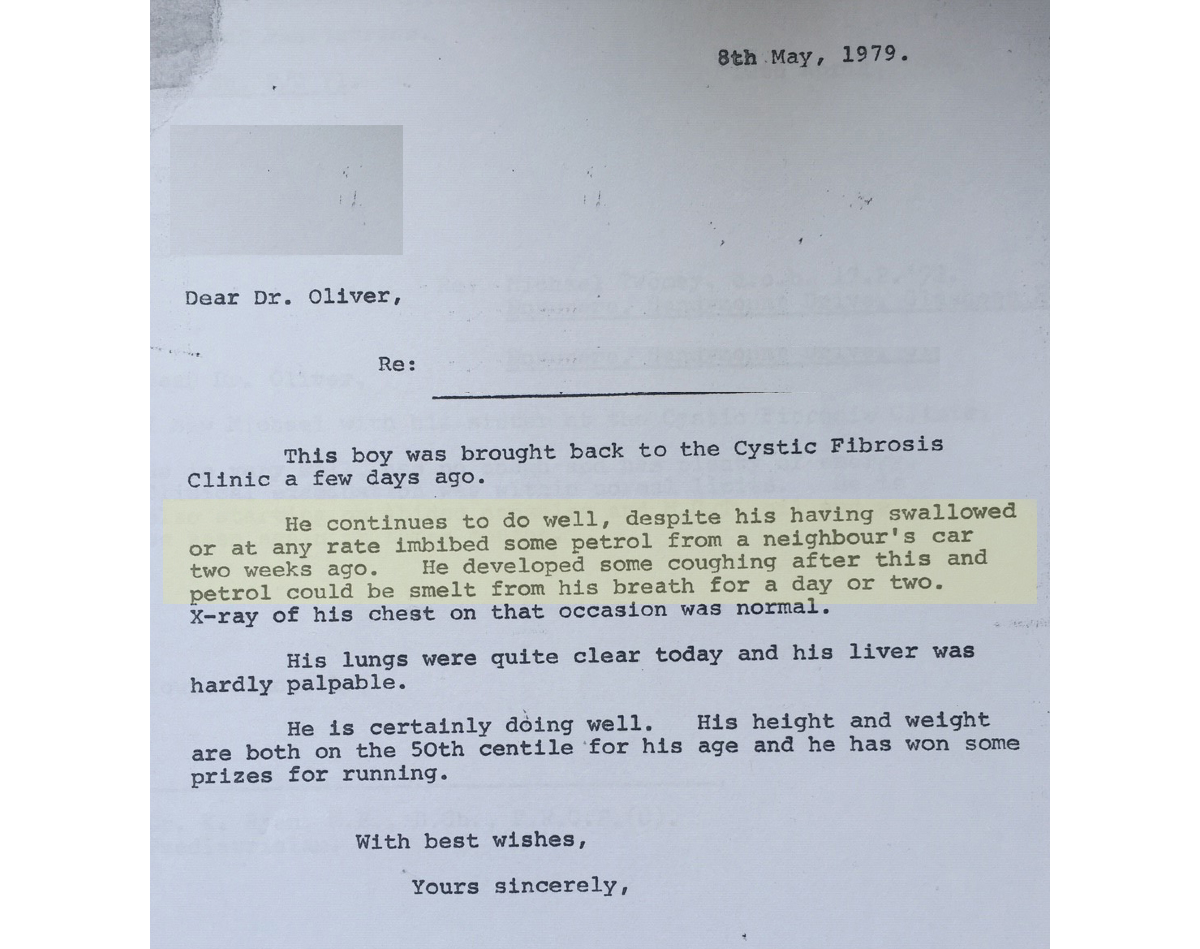
The petrol episode.

My appetite for learning and wanting to help other patients with CF and their caregivers was really kindled in October 2014 when I returned to postgraduate executive education, and it continues to burn brightly. For those who know me best, it probably comes as no surprise that I have become a researcher. Even at an early age, I was quite inquisitive and sought to explore and understand the world around me. However, it may surprise you to know that my first attempt at research failed miserably. I was just 7 years old, and I was trying to make sense of how one could get a liquid (in this case, petrol) to rise in a tube. I thought it fascinating until I imbibed or inhaled a gulp of it and also flooded the neighbor’s driveway. I was the talk of the neighborhood for about a week. Thankfully, it did not end in complete disaster, nor did it quell my inquiring mind, but I am sure I scared my poor parents half to death. This event became known as the *petrol episode* (Figure 2, in yellow).

It is a day that we at home do not speak of too often, except to remind me that I am capable of some awful blunders and that research does not always go according to plan. However, I like to think that that day in May 1979 gives you the reader a little glimpse of the character that I am, spirited, curious, and not afraid to explore!

Many would contend that, as a patient with CF with over 47 years of experience living with CF, I was the perfect candidate to research or explore and understand the associated problems of memory recall within the medical appointment. And yes, there is no doubt that I had the ability to impart rich CF-related information to the project; however, quite quickly, I became very cognizant of the fact that my illness journey (although sharing similarities with others) was my own unique voyage, with all the biases of any individual. Recognizing this and taking advantage once again of the robust ties that I had within the CF community, I enrolled 2 caregivers of patients with CF into a design team and a clinician specializing in CF to reduce the bias that I brought to the ADR project and to enrich the knowledge of the collective.

My patient-inspired investigation was driven by what eventually became the following motivation: how might I augment the memory recall or information retrieval of patients with CF and their caregivers within the elicitation and elucidation phases of the medical appointment?

Having attained ethical approval from the University College Cork Social Research and Ethics Committee, my primary focus eventually (discussed later) became the design of a checklist artifact over a 10-month period (September 2016 to April 2017). A checklist that would not only ameliorate the challenges of memory recall within the CF medical appointment but also augment our actual comprehension of these same challenges.

My research activity occurred in 3 iterative ADR cycles. Each iteration comprised rigorous naturalistic evaluation, involving subjective ex post interviews between myself and the evaluation team (7 adult patients with CF and 11 caregivers of children with CF), regarding their use of the artifact within their real-life medical appointments. In these evaluation interviews, I used qualitative metrics evaluating completeness, usability, robustness, and impact (Multimedia Appendix 1) [43], which helped shape our sensemaking process.

In tandem with this, I also sought expert opinions from clinicians on their appraisal of the checklist design and its subsequent use by patients with CF and their caregivers. These activities were followed up with learnings, reflections, and frequently additional consultation with the literature, followed by conformity by the design team on the design enhancements to be executed in any subsequent iteration, incorporating the intervention strategy of the same.

I now visit the next section of the paper, starting with the model of reflection I opted for, and the reflection (through 4 vignettes) on *problem formulation* within my ADR journey and its significance to efficacious outcomes.

## Reflection on My Action Design Research Journey

### Model of Reflection

In 1988, Gibbs [44] argued that it was “not sufficient simply to have an experience in order to learn; without reflecting upon this experience it may quickly be forgotten, or its learning potential lost. It is from the feelings and thoughts emerging from this reflection that generalisations or concepts can be generated. And it is generalisations that allow new situations to be tackled effectively.” After all, “we learn from reflection on experience. Reliving of an experience leads to making connections between information and feelings produced by the experience” [45].

**Figure 3:**
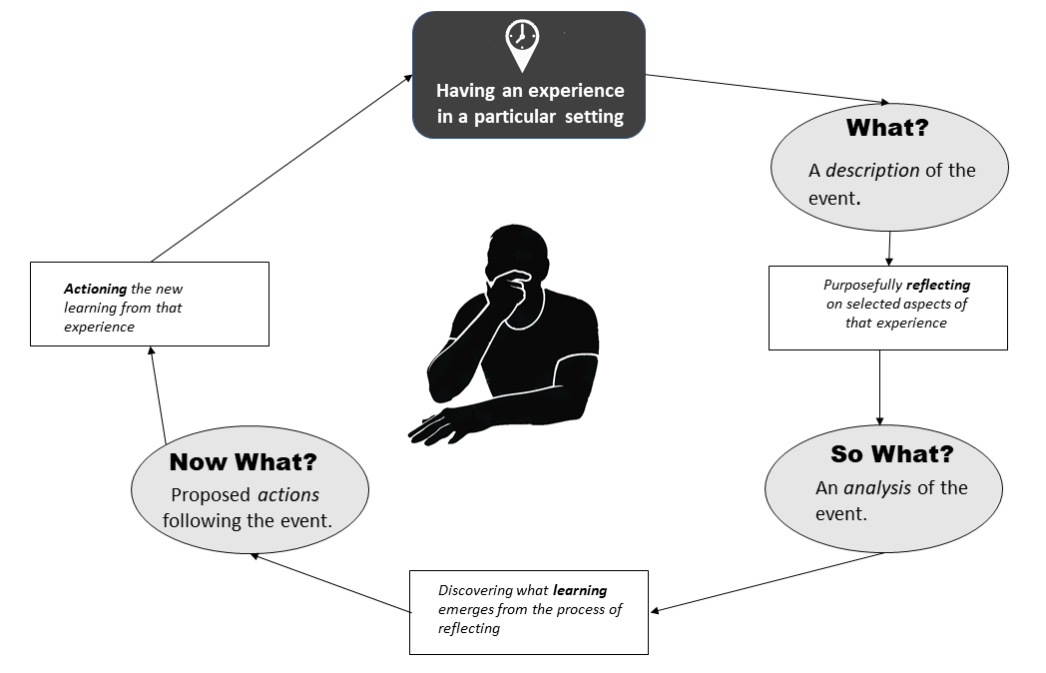
The What? Model of reflection - adapted from Driscoll.

Many of the seminal works on reflections or reflecting served as the initial stepping stone of my reflection in this paper. Although many models exist as possible viewpoints from which one might reflect, I opted to use Driscoll’s What? Model of reflection [46] (Figure 3), as I felt it resonated with me the most as an instrument to steer my reflection through the often-murky waters of my inner self.

Furthermore, as a reflection can be very personal and tacit in nature, it can be quite a challenging exercise or experience in and of itself. For example, it can take considerable time and may be painful and may even create a crisis of confidence [47]. That being said, it “offers distinctively grounded opportunities to pursue the connections between biography and social structure” [48]. Moreover, “reflection allows us to draw conclusions about our past experiences and develop new insights that we can apply to our future activities” [49].

Therefore, I felt it important to select the correct lens for me, one that would facilitate the recapture of my real-world experience rather than curtail or hinder the narration of my ADR journey as a patient and researcher. I reflect (using a series of 4 vignettes and Driscoll’s model) on an aspect of my ADR journey that I feel quite passionate about, that of the *problem formulation* stage of ADR, and of course its value to the fruitful outcomes of my study. I have also added a lesson learned in each vignette; this is merely to reinforce the key message I wish you, the reader, to take away from my meanderings.

Although my ADR journey is still ongoing, the paper-based checklist aspect of the project, the majority of which took place over a 10-month period (from September 2016 to April 2017), culminating in the creation of a checklist booklet in January 2019, is for now complete. It is this period that I wish to reflect upon, a *reflection on action* or a reflection through review as described by Schon [50], the process of review to inform learning [50].

**Figure 4:**
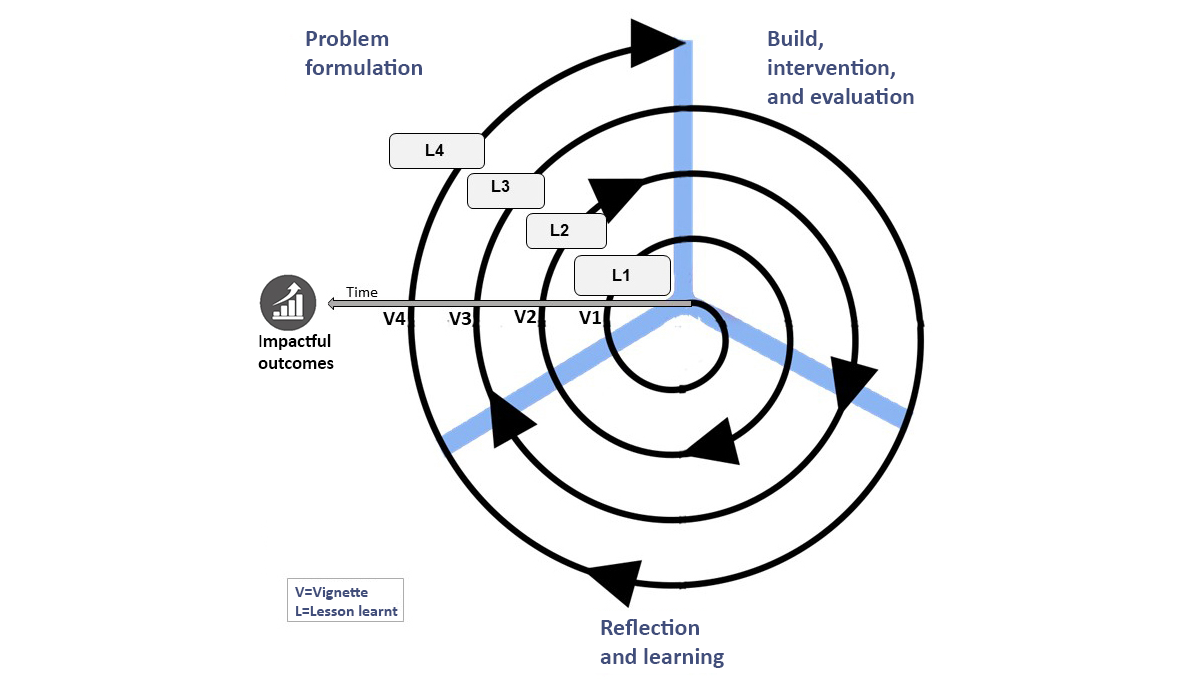
Visualisation of reflection within the context of the ADR project, where V=vignette and L=lesson learnt.

However, it is also important at this juncture to accentuate how and where these vignettes and learnings arose in the context of the overall ADR project. To this end, I include a simple diagram (Figure 4) that depicts the vignettes and learning in the context of the emergent, cyclical nature of the ADR project and its eventual outcomes. Although this reflection focuses on the *problem formulation* stage of ADR, it is critical to never lose sight of the fact that this stage moved in tandem with the other key stages of ADR.

These stages of ADR are akin to dancers performing the Tango, moving together in a closed embrace, individually and yet as one, influencing each other, each receptive to the other’s movements, shaping and being shaped by each other, all to a combination of an on-and-off-beat rhythm. It is in this spirit that I wish the reader to embark with me on my ADR journey. Although I try to minimize the use of any references, where I have used them, it is merely to enforce or enrich the musing of my ADR journey. And so, we move to my first vignette.

### Vignette 1: Tragic Thursday—September 2016

#### What Happened?

It was September 8, 2016. I was giving a presentation to my class on my efforts to date. I stood there like a proud peacock, chest out, boldly claiming the problem statement to be as follows:

There is currently no Patient Electronic Medical Records (PEMR) System that caters for the needs of CF patients or caregivers.

Therefore, patients with CF and their caregivers needed an app to manage their medical data. What is more, my design team and I were going to deliver it. We had created a number of wire-frames for each screen of the app, and here I was presenting them, “at long last CF patients and caregivers would no longer struggle in their medical appointments when asked by physicians about their medical histories” I said. Moreover, “when they leave the medical appointment, they will not struggle to remember the information imparted to them by the physician, it will sit on their phones and tablets and eventually in the cloud.”

And then it came, “I’m not convinced, I don’t think they will use it, I don’t think you have a handle on this yet” my innovation lecturer said. It was like he had pulled the rug from under me; I stood there shocked! The conversation that ensued between me and the lecturer (I am embarrassed to say) got somewhat heated, and what made the whole situation worse was it all happened in front of the entire class!

I returned home; the day had not gone as planned; I was very frustrated and quite upset; I had been publicly challenged regarding my solution and indeed the problem I thought I was trying to solve. I was now sitting in the kitchen with my head in my hands; I was not in a good place. Why was I having such difficulties with the *problem formulation* stage of ADR? My wife and child came into the kitchen and knew by my demeanor that all was not well. “What happened, did the presentation not go well?” she asked. “No, it was awful” I replied. “Let’s talk later” she said with a quick glance at our little boy.

Later on, that evening, I proceeded to give her all the gory details of what had happened. However, I did not realize my little boy (of 7.5 years) was listening as he had left the kitchen and had gone upstairs to his bedroom before the regurgitation of my day. However, as you know, children occasionally have a happy knack of overhearing or eavesdropping on discussions that they should not be privy to. This, despite our best efforts to protect them from the trivialities of adult conversations, and so, after ascertaining the gist of what had happened to me, he enters the kitchen with the swagger of a man who was going to expound some wonderful insight, and says “Daddy wouldn’t you think that after all the trips to the doctor that you have had, that you would understand what happens at a medical appointment?” I was left dumbstruck. I now refer back to that particular day as *tragic Thursday*, and yet on reflection, there was nothing tragic about it, in fact, the complete opposite is true; from that day onward, my eyes were going to be opened! I was going to realize the importance of this day as a turning point in my research.

#### So What?

I refer to the earlier event merely to illustrate my toil within the *problem formulation* stage of ADR. Although Sein et al [5] outline this as the first stage in ADR, they, unfortunately, do not refer to *how* one might or should go about *problem formulation*. I, probably like many others before me, ran into the solution space, convinced that I understood *the problem* that needed to be solved*.* I really thought after the presentation *on tragic Thursday* that I was the issue and that I may not be the patient or researcher for the job in hand. So, was it just me?

Well, yes and no. Let me clarify; as the earlier background section on *problem formulation* exemplifies, there is a lot more to *problem formulation* than one might initially think. Unsurprisingly, as we saw, we humans are not always logical in how we approach problems. We hold many biases and repeatedly make suppositions becoming fixated on unjustifiable assumptions [30]. These faults within humans often run counter to the successes that we strive to achieve and to the challenges that we strive to overcome.

Furthermore, solution fixation often results, leading to adverse consequences, precluding or hindering in-depth questioning or interrogation of problems, and prematurely freezing a problem space before it can fully form. I think the most unfortunate negative effect of poor *problem formulation* is on the divergent exploration of the creative possibility in design. I was an exemplar of this behavior, as a patient living with CF for 48 years, with countless visits to physicians, I thought I knew the problem *inside out*. As far as I was concerned, I had conducted an initial survey of 305 patients with respiratory illnesses and their parents back in 2015, and 77.9% (280/359) said they had difficulty in remembering their medical history. Furthermore, over 95% of them said they would use a secure app if one existed. I had taken offense to being challenged. I remember thinking, *what the hell does he know?* My ego had thwarted my ability to hear and appreciate the very sound advice that was actually being imparted to me.

Thankfully, I am not a stubborn fellow, and sense prevailed. I have since become a lot humbler and more open to criticism. I suppose, as a patient who has faced the question of my mortality at a very young age, I have become somewhat resilient, accustomed to picking myself up, dusting myself down (this often involves giving myself a good telling off, including the words—“*stop feeling sorry for yourself, remember why and who you are doing this for*”) and getting on with it. After all, someone had to resolve the issue, and if not me, then who?

Moreover, had *tragic Thursday* not happened, I would be sitting here on this Saturday afternoon in February 2020 (like many others before me) with a failed app. I most definitely would not be boxing up a solution for departure to the Royal London Children’s Hospital, London. A solution that, in the coming weeks ahead, will grace the laps of caregivers all over southeast England, within their real-world medical appointments.

#### Now What?

As we established earlier, I was not alone in my thoughts and tribulations regarding *problem formulation*. Mullarkey and Hevner [10] and others had also reported complications with this stage of ADR. My challenge then was to be mindful of the real need for an in-depth implicit problem analysis and to understand and define the information problem that patients with CF and their caregivers face during their medical appointment in tandem with a solution/s to ameliorate the same. My next design workshop was calling.

My first lesson learnt (L1) was as follows: If we wish to achieve successful outcomes, *problem formulation* requires a conscientious focus on problem comprehension, avoiding *solution fixation* and other assumptions.

### Vignette 2: So, What Is the Problem Again? —September 2016

#### What Happened?

I was sitting in the car in mid-September 2016. I was on my way to a workshop, looking forward to working with my design team, I was excited but also extremely nervous, it was only a week ago that my ship (I call the patient innovator) had taken a flurry of shots across the bow and nearly sunk, joining all the other vessels who had failed to survive on the wicked high seas in the world of innovation.

Many thoughts had been incubating in my head since *tragic Thursday*. My metacognitive processes were working furiously; my thoughts were a mix of emotions and ideas; to anyone brave enough to look inside my head, it would look *really messy*, like my room when I was a kid. I replayed the many medical appointments that I had attended in my mind, in tandem with all the literature that I had read in the area since autumn 2014. Although I had identified a problem or anomaly in the medical appointment, the issue that I now endeavored to solve was to understand and define the problem that patients with CF and their caregivers face during their medical appointment and delineate it in a way that made sense to me and others. Suddenly, a memory popped into my mind, vis-à-vis the time I endeavored to explain the issue to my darling wife for ≥10 min, after which she turned around and said, *so,* “what’s the problem again?” Moreover, I needed to be able to classify and represent the problem in a way/s that would assist my design team and I to see how we might best deal with it.

I was struggling; my experiences as a patient with CF alone were not sufficient to articulate and solve the problem. I felt as if my mind was constantly being polluted by irrelevant details and assumptions. I could feel my heart beating faster; questions flooded my mind; how was I going to structure or represent the problem? How broad was the problem? What were the constraints? What knowledge was needed to understand and solve it, and what gaps existed in my or our current knowledge base? What external and social factors would come into play? What strategy would we adapt? What did success look like? It was going to be a thought-provoking, challenging workshop!

#### So What?

Let us be honest; we all face problems of one kind or another every day of our lives. A 1-year-old may face the problem of how to stand unaided, or how to escape from their cot, the Alcatraz of their world. On the other hand, teenagers face the challenge of living in the evolving world of social media, acceptance, bullying, and so on. Problems come in all forms, some are simple, some are quite complex in nature, and others have an undeniably *wicked* composition.

Although there is a myriad of difficulties within the medical appointment, I needed clarity regarding the specific enigma (or part thereof) that I was going to focus on. I needed to avoid or at least be aware of the symptoms that were polluting and confusing the situation I was trying to remedy. Those related to other problems and yet overlapped with mine; otherwise, my ability to make sense of the issue with my design team was going to be a long, arduous process, one which would most likely end in failure. My design team and I needed to find an appropriate representation of the problem we had identified within the medical appointment, one that would give us insight/s to an appropriate solution pathway. Although my experience was beneficial and useful, it was only one CF patients’ voyage and nothing more; there were many more patients with CF and their caregivers that also had their story to tell, stories that would enrich our comprehension of the problem space. However, how should I go about gathering such insights?

The problem I found with the ADR methodology is twofold; first, it appears to hold a rather technocentric view of innovation; by this, I mean it does not seem to take into account the often-idiosyncratic nature of humans. This is evident in the lack of guidance on *how* we might or should come to truly understand the people behind a problem. For example, Who has the problem? Why is it a problem? What do they think? What really matters to them? What do they feel as they toil within a problem space? and so on. The ability to garner such fundamental human insights is crucial to disentangling, understanding, and defining a problem. Second, not enough emphasis seems to be placed on the *problem formulation* stage of ADR. It is almost portrayed as if *problem formulation* is a straightforward process, when in reality, the opposite is often true; it is frequently wicked and ill-defined.

#### Now What?

Arlin [51] argues that for a problem to be real, there needs to be a *felt need* to eradicate any impediments to an objective. Considering these words further, they conjure or evoke thoughts of sentiment, of emotions, the very essence of what makes us human. Therefore, to pursue ADR within the context of the social environment of the medical appointment, I also needed a deeply human-centered mindset, an approach that was profoundly human in and of itself. Unfortunately, I found ADR wanting in this regard.

In contrast, design thinking focuses on a user’s experiences and the emotions that are encapsulated in such events. Design thinking is a human-centered design methodology that “relies on our ability to be intuitive, to recognize patterns, to construct ideas that have emotional meaning as well as functionality, to express ourselves in media other than words or symbols” [52]. As one might imagine, some of the core principles of design thinking are empathy with users and discipline of prototyping.

Emotions are an integral part of what makes us human. Therefore, my design team and I began a series of design thinking workshops, beginning with a number of design thinking tools, from personas (Multimedia Appendix 2) to empathy maps (Multimedia Appendix 3) and journey maps (Multimedia Appendices 4 and 5). This meant spending a great deal of time with fellow patients with CF and their caregivers discussing the many facets of living with CF, their experiences (building on mine) within the medical appointment, and capturing their reality of being a patient with CF or having a child with CF, which are unique perspectives that were both profound and deeply insightful and often times quite moving. Empathy became the key to helping define the problem, but why empathy?

Many different interpretations of empathy exist from sharing the feelings of others and comprehending the emotions of others [53-56] to feeling what another experiences and the ability to appreciate the views of others [57]. Although we shared a medical condition, our life journeys are our own, unique, molding us, and shaping us into individuals. I came to understand the experiences of other patients with CF and their caregivers, assisting me to feel what they felt, I came to comprehend the stress they experienced living with CF, something that they (and I) have learned to manage daily. I heard of their experiences of holding a conversation with physicians while being short of breath, described by an adult patient with CF:

I was gasping for air, and trying to remember stuff for the doctor.

A young mother of a child with CF explained to me what it was like trying to list the medications that her child was on at a particular medical appointment:

My 5-year-old child was really sick with a chest infection, she was crying due to the pain in her lungs, it was impossible to concentrate, it felt like I had 500 things going through my head, I was so stressed. I remember thinking, what if I make a mistake? What if I leave something out? I felt so guilty and helpless at that moment.

Although insightful, I confess that, at times, I found this aspect of the project very difficult from a personal perspective. However, I learned to manage my own feeling quite quickly, forcing myself to compartmentalize my thoughts and feelings when and as required. I knew that this was critical if I wished to succeed and avoid floundering on the rocks in the sea of my own internal thoughts and emotions.

Nevertheless, it is important to note that it was through this appreciation of the problem through the senses and experiences reported by other patients and caregivers that we (my design team and I) would come to realize that a technocentric approach to the problem was not an appropriate starting point. It was only by really listening to what the patients and caregivers were telling us that we came to grasp what we were really dealing with. They were not, in fact, talking about apps or technology at all; they were complaining about their needs regarding their information and their frustrations regarding access to their medical history when and as required. A patient explained:

If I’m away traveling, I need to have my medical information at hand in case I get sick. I can’t walk into a doctor who knows nothing about me or my condition. This happened to me before and I was put on the wrong treatment. It was very upsetting; I could have died.

My design team and I eventually settled on the following new problem statement: *“*CF patients and caregivers are not having their information needs adequately addressed within their medical appointments.*”*

Interestingly, renowned cardiologist Eric Topol [58] argued that “patients exist in a world of insufficient data, insufficient time, insufficient context.”. In line with the principles of design thinking, we decided to engage in pretotyping (paper-based prototyping) in the form of a checklist to aid in our comprehension of initial interest and actual usage by users of our solution. The pretotype was designed for patients with CF and their caregivers to fill out before and during the physician’s appointment. Pretotyping (conceived by Alberto Savoia [59]) also made sense as it enabled the smallest investment of time and money possible, while still facilitating the capture of distinctive insights from users of the checklist within the context of the medical appointment. The checklist pretotype was also prudent as a precursor to any digital solution that we may eventually embark on. It also steered me clear of *falling in love* with any early solutions. I wished to avoid another tragic Thursday. Checklist iteration 1 was designed and released at the end of September 2016 (Multimedia Appendix 6) [43] with evaluations conducted at the end of October and through November 2016 (Multimedia Appendix 1) [43]. Interestingly, even at this early stage, the checklist demonstrated positive outcomes, with 81% of participants reporting better memory recall as a result of using the checklist.

My second lesson learnt (L2) was as follows: *Problem formulation* requires in-depth human-centric exploration—scrutinizing a problem thoroughly through the senses of those who experience it, understanding how it affects them, and culminating in the articulation of an accurate problem definition and more positive solution outcomes.

### Vignette 3: Teaching an Old Dog New Tricks—May 2017

#### What Happened?

**Figure 5:**
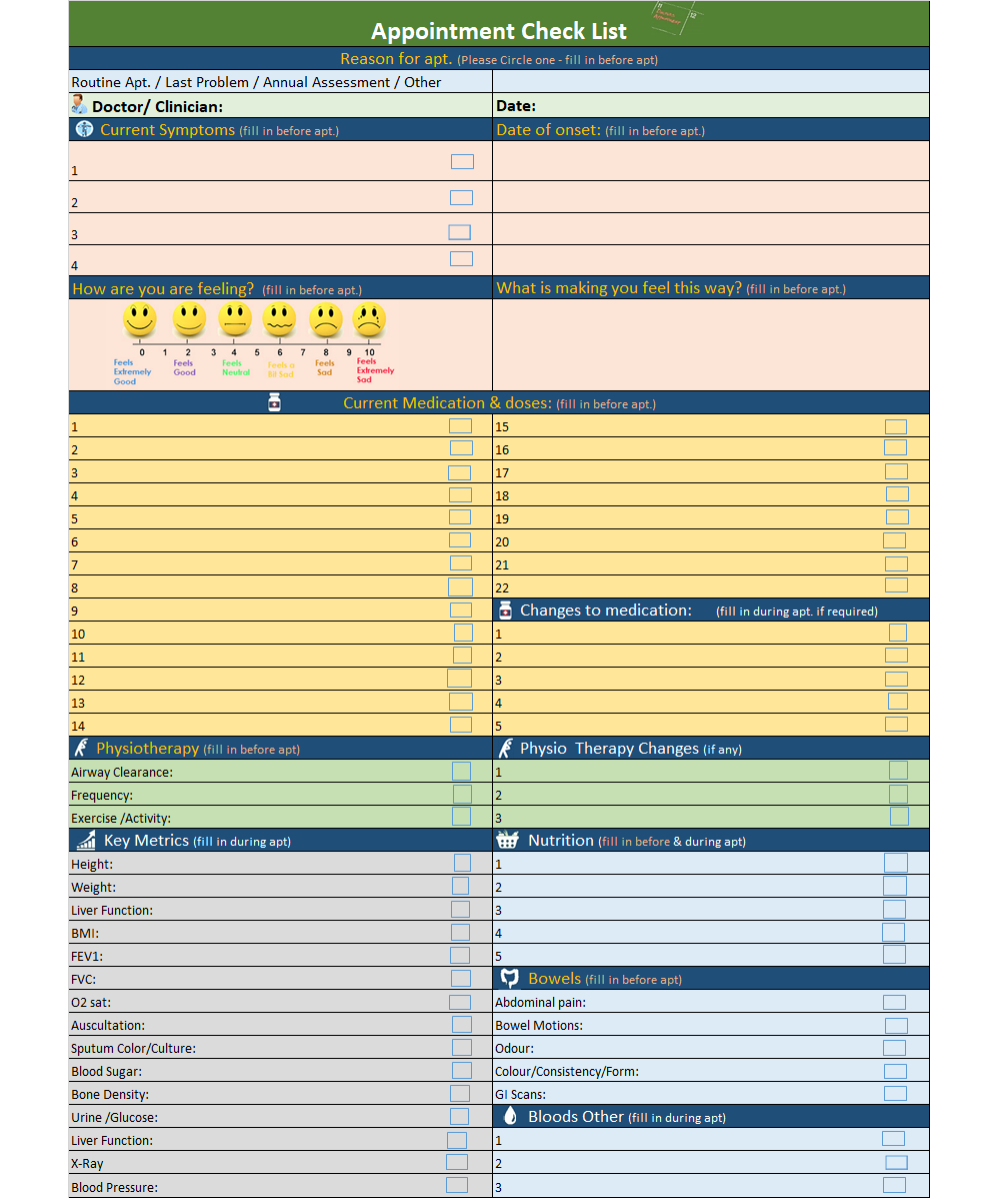
Version 3, part 1, of the checklist.

**Figure 6:**
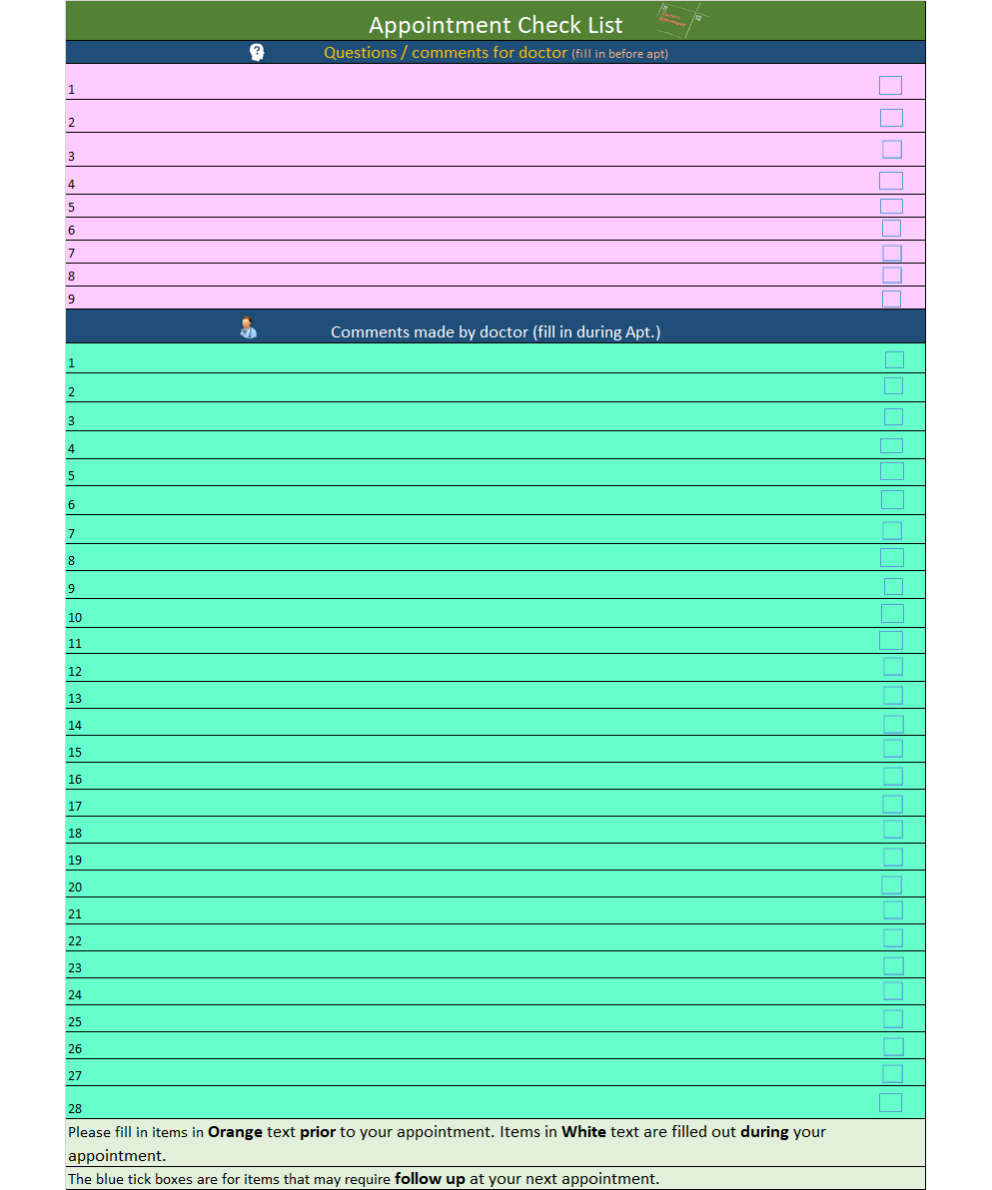
Version 3, part 2, of the checklist.

It was a beautiful morning in May 2017 and I was out walking my dog, *Suzy,* down in the local forest not far from home. There was a very light breeze, the trees gently swayed to the dawn chorus of bird acoustics, a melody of song and calls. I had recently completed iteration 3 [43] (Figures 5 and 6) of the checklist, and thankfully, the evaluations were very positive (Multimedia Appendix 1) [43], with the artifact having major impacts within the CF community. Here, patients and caregivers reported reduced stress and increased empowerment as a result of having their medical information with them during their medical appointments and, of course, afterward. The checklist really appeared to work, but why was it working so well?

What had we done that seemed to be tipping the results in our favor? Our enhanced understanding of the problem definitely seemed to be manifesting itself in the delivery of an improved solution? However, I had this innate feeling that I was missing something, that we still had not achieved an absolute sense of why the checklist was so impactful? I was bothered.

I proceeded down the forest path toward this small brook in which my dog was playing. She was play acting with a stick, when much to her dismay she dropped it, and it disappeared into some sort of small drain. I watched her with as much a sense of curiosity as amusement, as she endeavored to retrieve her prized possession. She approached the drain from one angle, then another, and then back again. This all went on for a number of minutes, it was clear from her expression that she was engaging the very limits of her cognitive abilities, as she tried to make sense of the predicament. Eventually, she managed to work it out, after several failed attempts doing it one way, she suddenly approached the problem differently, in a simpler fashion, she had her stick again!

My mind wandered back to my own thoughts, was there other ways that I should be tackling or viewing the information challenges that patients with CF and their caregivers were experiencing as well? Surely there were additional ways from which I could view the problem and solution, possibilities yet to be considered, ways that may come to enlighten me as to why the checklist was begetting such light into the often dark setting of the medical appointment with regard to memory recall. I was convinced that there was more to learn and more to understand, and yet, every time I tried to think about it, I found myself back where I started. It was like a neural impasse, as if the mental pathways in my mind were predetermined, immovable objects, defeating my abilities to explore new possibilities and new ways of thinking. I grew frustrated; my mind was tiring. I kicked a stone into the brook, “if only we had a more advanced memory, akin to some sort of futuristic form of Artificial Intelligence then there wouldn’t be this stupid problem” I thought. And then just like my dog a few moments earlier, a new thought entered my mind, “If we had perfect memories, we would not need a solution.”

The problem I thought was not really *information needs* as much as the limitations of human memory, arguably defective, often resulting in an inability to remember, a failure to recall memories on demand, and a malfunction of our biological information retrieval system. Inadequate *memory recall* was the real problem, it had been there under my nose all the time, and yet I failed to see it, until now, months later.

#### So What?

The abovementioned vignette reminds me of the American inventor and engineer Charles Kettering when he declared that “a problem well stated is a problem half-solved” [60]. So, what exactly did Kettering mean here? Do we take from his statement that once a problem is stated, we are halfway to a solution? Or does he infer something more arcane, that even with a well-stated problem, there is abundant knowledge yet to be unearthed, comprehended, and considered, regarding how and why solutions perform as they do within their intended environments? Having traveled through my ADR experience, I think he may well have intended for us to appreciate both in combination and individually.

In May 2017, I really realized and appreciated the richness of looking at a problem through multiple lenses. I was also amazed at how static my cognitive frameworks or schemas were. Moreover, I was amazed at how long I had stayed in these cognitive states, unable to move, paralyzed if you will, this despite numerous evaluation interviews with patients and caregivers and workshops with my design team. Why had it taken so long for the older mental model to be replaced and augmented with a new one that would enhance my explanatory power? Was it the assumptions I was making while trying to make sense of the complex environment of the medical appointment? Had I become locked in on a particular mental representation of what I perceived was the *sweet spot* of the issue?

I mentioned earlier that “what we understand and how we understand a situation depends on the information we bring to a given situation, and the longer we think about the situation the more its cognitive representation changes. It may be assumed that cognitive elaboration activates more schemata” [42]. I was fascinated by this and yet cautious, what other points of view had I not considered? In spite of these contemplations, I decided that I would refrain from tormenting myself and spoiling this moment in the process.

I had no doubt that as I continued on my voyage, I would discover new ways of thinking of framing my understanding. However, a sense of balance and perspective is required; one must avoid entering a state of *analysis paralysis*. As the esteemed English writer Samuel Johnson once said, “nothing will ever be attempted if all possible objections must first be overcome.” I would take each enlightenment as it came, relishing the cognitive challenge that each schema would bring.

More than one account of a complex system is achievable, where altered portrayals will break the system down in diverse forms, and changed descriptions may also have altered levels of intricacy. I was both relieved and excited that I had discovered a new viewpoint from which to perch my telescope of inquiry. Moreover, I felt an augmented confidence ignite within me, the cause of which was twofold; first, I now felt more assured regarding my grasp of the actual problem, and second, I sensed more confidence in my appreciation as to why the checklist was functioning so well for patients with CF and their caregivers.

#### Now What?

I relayed my thoughts to my design team, and we came up with a new problem statement: *The challenges of memory recall or information retrieval that CF patients and caregivers have or experience within the medical appointment are not well understood or solved.*

**Figure 7:**
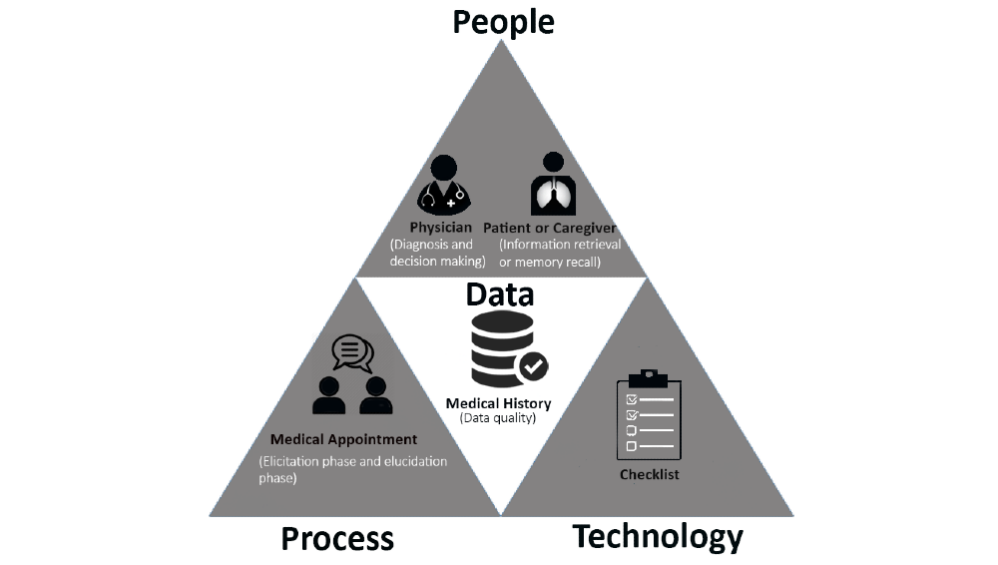
People, process, technology, and data.

This made a lot more sense to all of us, and so taking a people, process, technology, and data view, we created a model (Figure 7) to depict our new representation or understanding of the problem, including the roles of memory recall or information retrieval within 2 key information stages of the medical appointment. I have also expanded on each of these people, process, technology, and data concepts as they pertain to the medical appointment in Table 1. The usefulness of Figure 7 and Table 1 is twofold: first, to depict my interpretation of the problem, and second, to highlight the advancement of my comprehension of the various interacting or moving components within the problem space.

Moreover, I came to realize why the checklist was so effective, it was in effect acting as a tool aiding the long-term declarative memory of the patient and caregiver during their medical appointments. From another perspective, one could say that it achieved this by actually relying less on the patients or caregivers’ own memory and more on the checklist within the appointment. Patients and caregivers now came prepared with the information required within the elicitation phase of the medical appointment already written down in front of them. Moreover, they had a placeholder to capture key information within the appointment as well.

**Table 1:** People, process, technology, and data model concepts.

Concept				Reference
**People**				
	**Physician**			
		The purpose of the medical appointment is to “make the diagnosis.”	[61]	
		The consequences of poor memory recall or information retrieval are: On the quality of information imparted to a physician, the ability to make an effective diagnosis and treatment decisions, and impacts on patient outcomes.	[62]	
		Clinician satisfaction	[63]	
	**Patient or caregiver**			
		Research shows “that memory for medical history, like other forms of autobiographical memory, is likely to be flawed, incomplete and erroneous.”	[62]	
**Process**				
	**The elicitation phase**			
		Physician and patient or caregiver participate in a bidirectional conversation regarding the patient’s medical history, current well-being, current medication, and so on.	[64]	
		Furnishes the physician with 60%-80% of the data required to make a diagnosis.	[65-67]	
	**The elucidation phase**			
		Physicians communicate diagnoses, clinical options, and self-care plans, in tandem with overall advice regarding the management of a medical condition/s.	[68]	
		This phase directly impacts patient adherence and other self-managing activities, such as regime change.	[69]	
**Technology**				
	**Checklist**			
		Defined as “a formal list used to identify, schedule, compare, or verify a group of elements or...used as a visual or oral aid that enables the user to overcome the limitations of human memory.”	[70]	
**Data**				
	Medical history data includes the following: medical appointments, symptoms, illness episodes, encounters with other clinicians, medical therapies and medications.			[62]

Hence, they would not need to rely on memory when they left the medical encounter either. They had it all in the checklist and could refer back to it as required, even when traveling, if they happened to get ill. It probably comes as no surprise then why patients or caregivers were reporting such impacts on stress and empowerment. Additionally, I really came to fathom the potency of precise *problem formulation* vis-à-vis its impacts on actual outcomes. However, as one might expect, as I delved deeper into the area of memory recall during the medical appointment, I realized there was a lot more to this puzzle than I first envisaged or ever imagined on that beautiful summer morning in May 2017.

My third lesson learnt (L3) was as follows: In the *problem formulation* stage of ADR, we must challenge ourselves to look at a problem from different perspectives and from alternative disciplines. If we have not found or considered alternative viewpoints, we may fail to understand a problem well enough, affecting the most appropriate articulation of the problem definition, and the successful design of a solution or comprehension of why a solution functions as it does.

### Vignette 4: Breaking It Down—May 2018

#### What Happened?

It was a warm, humid day on May 25, 2018; the sky was cloud-flecked; and the various bird songs bestowed a pleasing accompaniment to the day. I was on my way to a symposium in my university to give a presentation on my research. I was nervous; my stomach was making noises, clamoring’s that I hoped were inaudible to the various scholars gathered in the room. I imagined that none would be too fond of hearing such clamoring ascend from my abdomen.

I hoped they would, however, be very interested in hearing how the checklist we had designed, built, and evaluated, functioned so well within the environment of the CF medical appointment. Moreover, following a 9-month rigorous systematic literature review of memory recall within medical appointments, I sought to impart where my comprehension of the problem had advanced to and why carrying out such an activity was fundamental to unlocking the additional knowledge I required in the *problem formulation* stage of our ADR project.

#### So What?

I had decided to conduct a meticulous literature review for 2 reasons. First, I wanted to understand why the checklist was functioning so well in the medical appointment. Second, I wanted to see if I was overlooking anything, for example, was defining the problem as memory recall or information retrieval of patients with CF and their caregivers within the medical appointment comprehensive or deep enough? I found that the answers to both questions were, in fact, deeply intertwined.

In the first instance, I came to understand that human declarative long-term memory was analogous to many complex systems consisting of components, in this case different memory types: episodic memory, autobiographical memory, and prospective memory (Multimedia Appendix 7 [71-77]). The components themselves are often simple (or can at least in this instance can be viewed as such) and interact with each other through various routes possible among components, mediated in distinct circumstances.

So why was the checklist functioning so well in medical appointments? Henry Ford is noted for saying, “Nothing is particularly hard if you divide it into small jobs.” Breaking down memory recall or information retrieval into its components, studying the physician and patient narratives (supplied by consenting patients and caregivers from real medical appointments), and assigning declarative memory components to each sentence or group of sentences allowed me to unearth a more profound comprehension of the complexities of dialogues within the medical appointment and the variety of long-term declarative memory components used therein. I also came to truly appreciate the pressures that memory recall places on the patient and caregiver, such as recalling a particular episode (episodic memory), time period/s (autobiographical memory), or remembering to report symptoms at an appointment (prospective memory) or a combination of declarative memory types. In addition, I found that the checklist design actually maps to *aid* the declarative long-term memory component drawn upon by the patient or caregiver during the medical appointment. This deeper, more comprehensive level of understanding of memory recall or information retrieval, breaking it down into its components, afforded me far deeper knowledge from which to view, examine, and indeed make sense of the problem I endeavored to solve, and of course as already put forward, why the checklist functioned so well for patients with CF and their caregivers within the complexities of the medical appointment.

Second, although a single checklist simplifies the capture of information at a particular medical appointment and, thus, aids the episodic memory of the patient or caregiver regarding that specific event, I came to understand that from an autobiographical memory perspective, the checklist was still somewhat inadequate. I wanted to support the autobiographical memory of patients and their caregivers in the best way possible, and, although theoretically, one could file away a single printed checklist in a folder after each medical appointment, I decided that this would not suffice. Patients and caregivers have enough going on in their lives without trying to find another workaround. Being a patient myself, I was sick to death of always having to settle for second rate solutions, solutions that I would later try and adapt to my own needs. On several occasions, I remember thinking, why is it that no one can get this stuff right? Are we (patients) that hard to understand, are our needs that difficult? Or is it that no one really gives genuine thought when designing products for us? I could not be a hypocrite; I had to improve the checklist, no workarounds!

#### Now What?

First, my design team and I came up with a new problem statement: *The challenges of memory recall information retrieval (and its components) that CF patients and caregivers have or experience within the medical appointment are not well understood or solved.*

**Figure 8:**
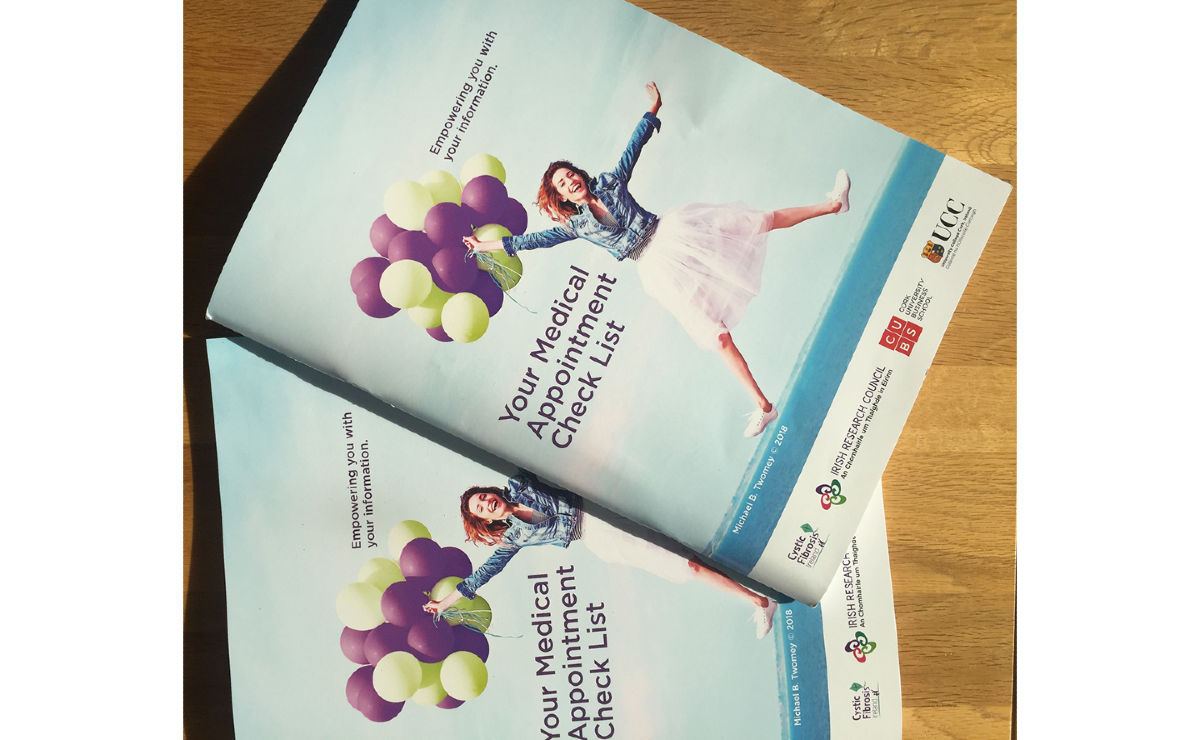
The checklist booklet.

Second, after iteration 3, and my comprehension of long-term declarative memory components, we decided to create a professional, physically robust booklet (Figure 8), with a little help from a professional graphic designer (a friend of mine; Multimedia Appendix 8 shows the final checklist). A booklet containing 28 checklist items, unshackles the patient or caregiver from having to do any workarounds, including any printing. At once, a repository of medical discourse is created, where 28 medical appointments checklists are held together, not only facilitating the episodic and prospective memory of patients and their caregivers but also acting as an autobiographical memory of a specific time span. Searching for a previous appointment/s was now simple and straightforward.

Many may argue that this further exploration was a step too far, unnecessary, and indeed prohibitive for many researchers and organizations in terms of cost, and so on. Although I appreciate these sentiments, I would not agree. If you really care about the user’s experience (and you should) and you want to deliver quality impactful solutions, you must be willing to *go the whole nine yards.* In fact, I believe this is the only way to accomplish truly successful outcomes. To do anything else is to cheat yourself, your organization, and, most importantly, the user from what might have been.

Had I not gone deeper, the checklist as a booklet would never have come to pass. Granted, I would have a checklist (as a single page); however, I cannot say I would be confident that a patient or caregiver would continue to go to the trouble of printing a checklist before every appointment, and then file it away afterward. One must appreciate that patients with chronic diseases and their caregivers are busy trying to lead as normal a life as possible, and they often have very complex and time-consuming treatment schedules. Hence, asking what may seem a simple task can, unfortunately, often be the *straw that breaks the camel’s back* for a patient or caregiver and hence lead to unused or underused solutions. By having a deeper grasp of the problem, I was able to put this knowledge to good use, advancing to a more robust solution. This, I believe, is why so many are now requesting the checklist booklet.

Additionally, when I explain the rationale behind the workings of the checklist booklet to those with the CF community, including clinicians, I can see that they are really intrigued, and if I may be so bold as to say, excited by the solution. This has of late manifested itself in a large amount of dialogue within the community, much of which I am only now becoming aware of, as it has translated into invitations to various clinical conferences, and international requests to see the checklist booklet, and of course boxes of checklist booklets being taped up on a Saturday afternoon. Checklist booklets that will soon find their way into the hands of patients with CF and their caregivers far and wide.

My final and fourth lesson learnt (L4) was as follows: *G*oing deeper in the *problem formulation* stage of ADR will help to explain observed phenomena, highlight shortcomings in a solution, and enrich problem definitions, resulting in a truly comprehensive understanding of a problem domain and the delivery of truly successful impactful solutions.

## Concluding Remarks

**Figure 9:**
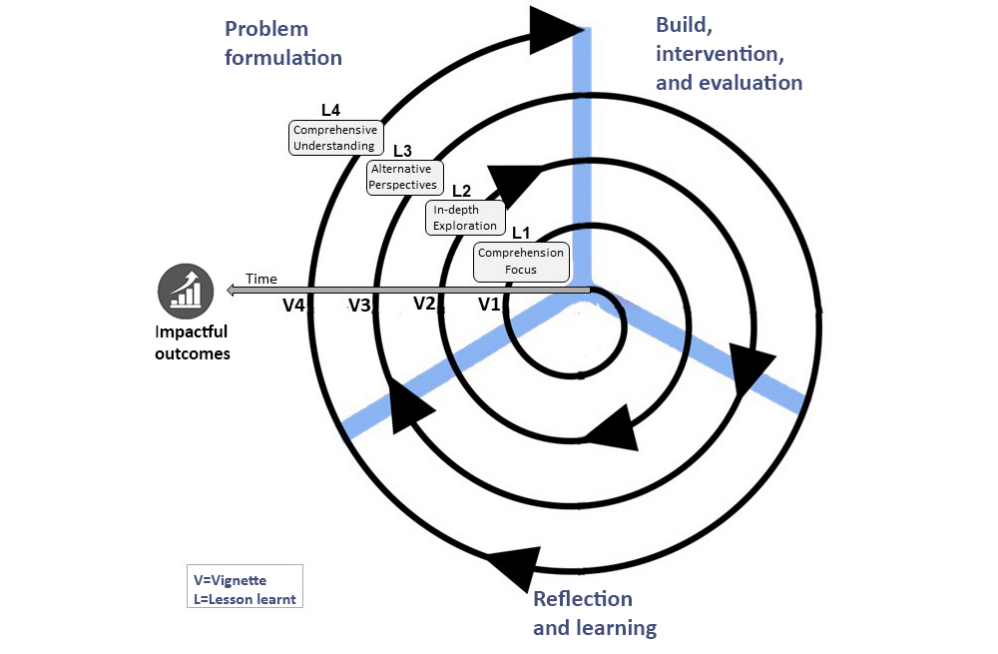
Visualization of reflection within the context of the action design research project.

Unfortunately, that a small amount is appreciated vis-à-vis how problems are formulated in ADR seems as true today as it was six decades ago. In many of the ADR papers that I have read, there appears to be a very quick shift in focus to the subsequent stages of the methodology, with little mention or focus on the *problem formulation* stage. Moreover, *problem formulation* is seldom mentioned again in manuscripts. This is despite the iterative nature of ADR (Figure 1), where after *reflecting and learning,* the researcher/s refer back to the *problem formation* stage to ascertain whether or not a problem definition has changed or evolved. This is, of course, not to say that the stages that follow the *problem formulation* stage are not important, quite the contrary, they are also fundamental to an ADR project. Hence, I have included the same again in Figure 9, which, unlike Figure 4 earlier, now includes the lessons learned (as concepts) from each vignette extracted from my ADR journey. You will note in Figure 9 that our impactful outcomes are only attained at the culmination of our journey through iterations of ADR and *problem formulation* exploration and determination.

The question that I still contemplate is, why further regard is not given to this crucial stage of ADR? In my researcher story overlaid on an ADR story, I have bought to bear (through my series of vignettes) how difficult this stage of ADR actually is. I have also tried to portray what can go wrong without a conscientious focus on problem comprehension. Moreover, I have highlighted how beneficial time spent in this stage of ADR is, in terms of research impacts and results. Surely, I am not alone in my struggles as a researcher in *problem formulation*?

As I have already stated, but wish to emphasize once more, it is fundamental to empathize or understand the people behind a problem, what they experience, what they are feeling, and what and how they think, if you wish to deliver truly impactful solutions and sought-after outcomes. Indeed, Southard [78] was the first to articulate the significance of empathy in the physician and patient therapeutic relationship and its role in assisting diagnostic (*problem formulation* within the medical appointment) outcomes. To do otherwise is to deny our humanity, blocking the very comprehension we require to address the often-difficult problems we encounter, as we go about our lives on a daily basis, navigating the many complex systems within which we live.

In vignette 3, I contended that we challenge ourselves to look at a problem from different perspectives, to be more open to other disciplines, and to be prepared to “repeatedly change our point of view, our way of looking at the problem. We have to shift our position again and again” [79]. This augmented comprehension not only facilitates a more appropriate articulation of a problem but should also liberate insights into why a solution functions the way it does within a particular environment.

However, as illustrated in L4, we must also be willing to probe and dissect these new viewpoints further, atomizing them down into components and concepts, facilitating deeper insights into phenomena, deepening our problem definitions, and thereby enabling a truly inclusive augmented comprehension of a problem. Additionally, such curiosity draws our attention to inadequacies in our solution/s, such as the checklist before it being in booklet form, where it really failed to address autobiographical memory. Through this knowledge, we further enhanced the checklist. Without delving deeper, this would not have happened.

The checklist produced evolved and was shaped, not only by the environment into which it is placed but also by my mind, which also underwent a type of metamorphosis, as empirical findings and knowledge waltzed together to the beat of my heart and the passions and conviction that expounded from within. Time and time again on this ADR project, I felt like I was on a journey of self-discovery, with many twists and turns, good days and bad days, days that taught me some valuable lessons, experiences that sometimes had a real sting to them, but will not be forgotten.

None of it was, of course, in vain; quite the contrary, I know I have made a real difference and continue to change the lives of many patients with CF and their caregivers during their medical appointments. I could not ask for a better outcome in my research endeavors. As pointed out earlier, I put this down to both grit and determination, especially as I have shown, within the *problem formulation* stage of my research. Therefore, fundamental to ADR success is the continuous revisiting of *problem formulation* after each iteration of an artefact, it is only by doing same that we can hope to gain a truly augmented understanding of a problem, and become more confident in designing and in our solution designs. I hope what I have discovered and aimed to impart here proves useful and insightful to those who brave the high seas of *problem formulation* in ADR, helping them to avoid some of the fatuous mistakes that I have made while on this chapter of my ADR voyage. Aiding them reach their intended research destination in one piece, confident that they too, have delivered impactful solutions through an augmentation of problem comprehension.

In tandem with the above viewpoints, I would advocate for the inclusion and portrayal of the actual realities of this stage (as I have endeavored to accomplish in this reflection) to be included and explored by researchers and practitioners. I feel that the insights garnered regarding same would not only bring a sense of realism and humanity to research (a component that I feel is often missing), they would also generate contributions to knowledge in and of themselves, the *how to* or *how I or we* navigated challenges encountered in research.

I miss my dear sister, but I choose to honor her memory by doing something that I know would bring a warm smile to her face. I will be honest at this moment I have no idea where my researcher voyage will eventually take me, but it feels so right. Like a faint whispering in my ear that gets louder each day, like a fog lifting, giving a clearer aspect to the road ahead. My heart quickens as my quest becomes clearer.

## Appendix

Multimedia Appendix 1 Checklist evaluation summary.

Multimedia Appendix 2 Personas.

Multimedia Appendix 3 Empathy map.

Multimedia Appendix 4 Journey Map - Activity Cycle.

Multimedia Appendix 5 Journey maps—Day in the life.

Multimedia Appendix 6 Version 1 of the checklist.

Multimedia Appendix 7 Declarative memory types.

Multimedia Appendix 8 Booklet version of the checklist.

## Abbreviations

ADR: action design research
CF: cystic fibrosis
DSR: design science research
IS: information systems
IT: information technology
