# Supplementary figures and images for: The Tango of Problem Formulation: A Patient’s/Researcher’s Reflection on an Action Design Research Journey
Source: J Med Internet Res. 2020 Jul 14;22(7):e16916. doi: 10.2196/16916 (PMC7388038; doi:10.2196/16916)

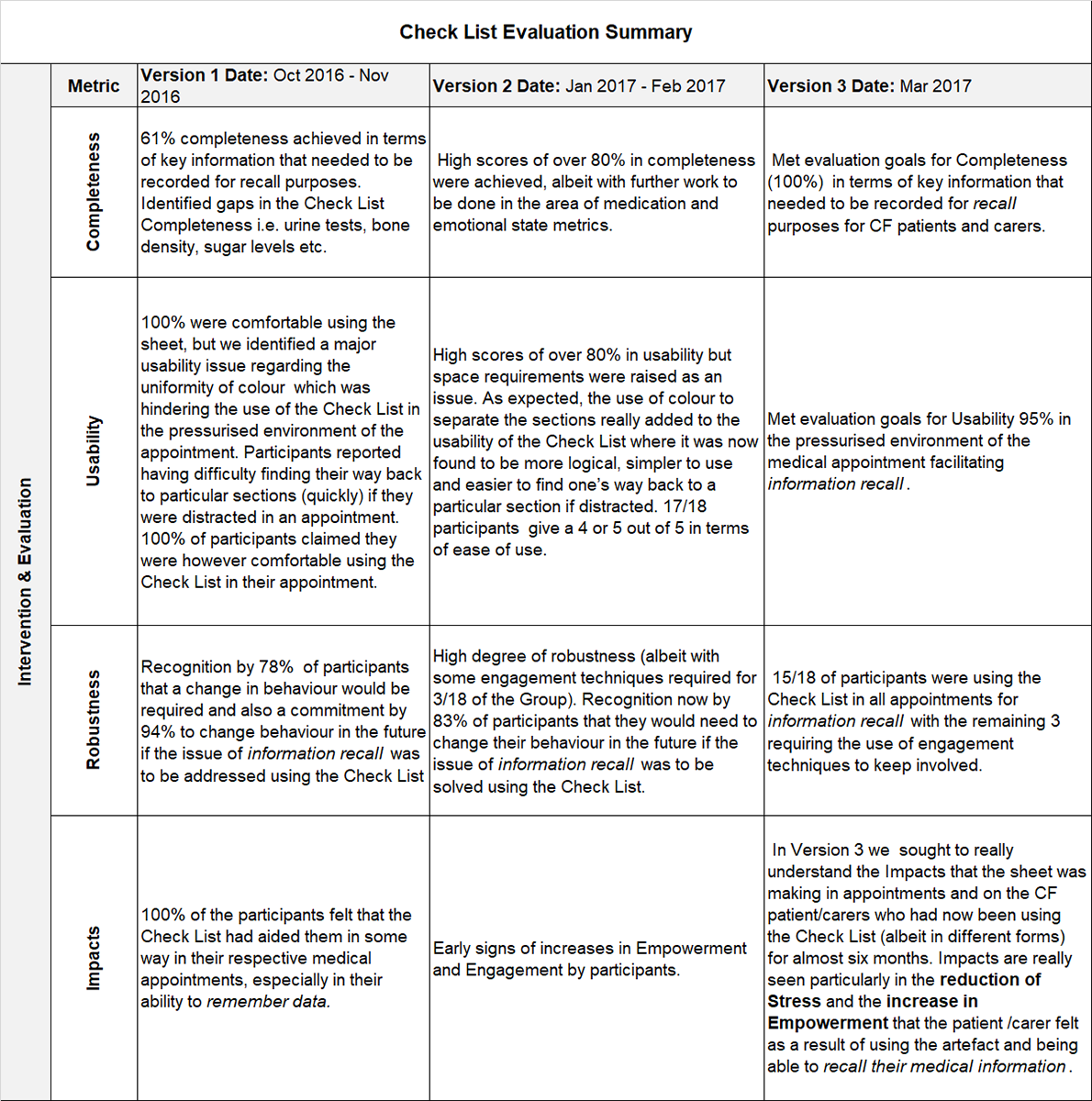

Supplement: Multimedia Appendix 1 [file jmir_v22i7e16916_app1.png]

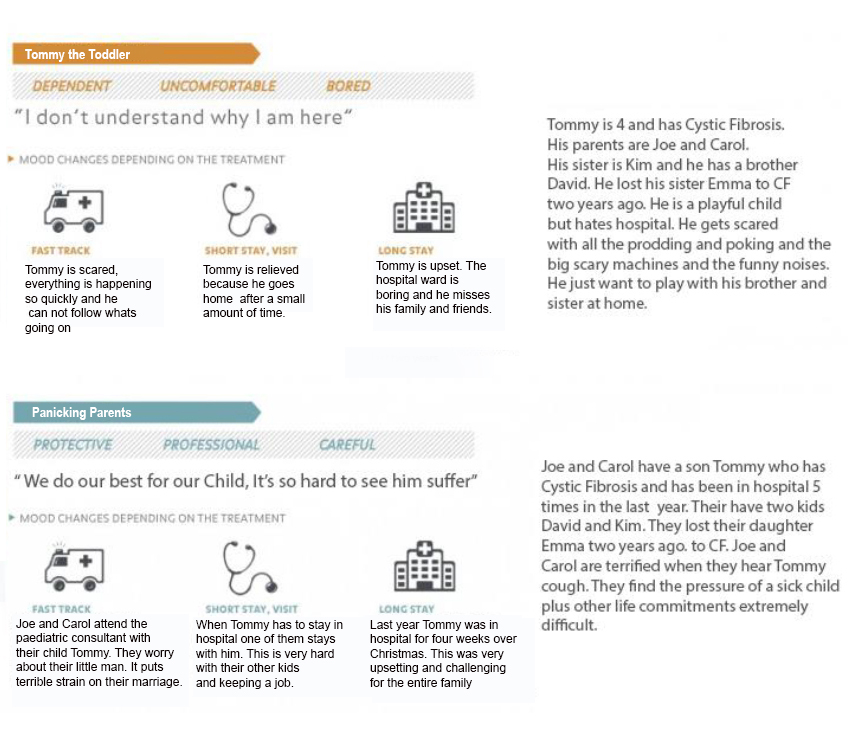

Supplement: Multimedia Appendix 2 [file jmir_v22i7e16916_app2.png]

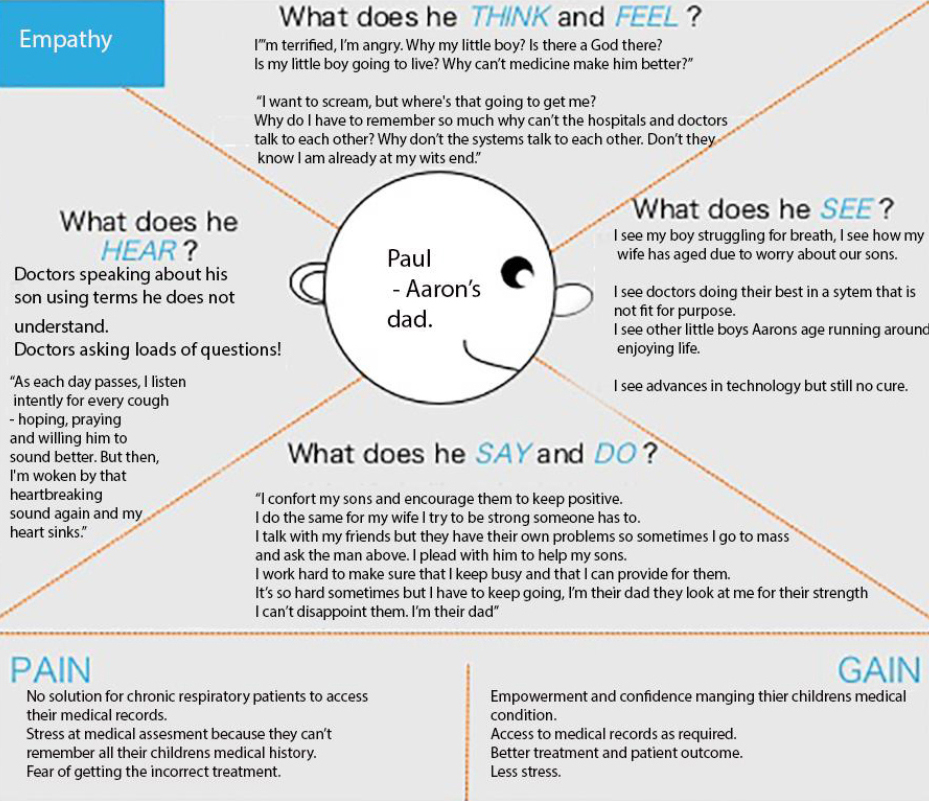

Supplement: Multimedia Appendix 3 [file jmir_v22i7e16916_app3.png]

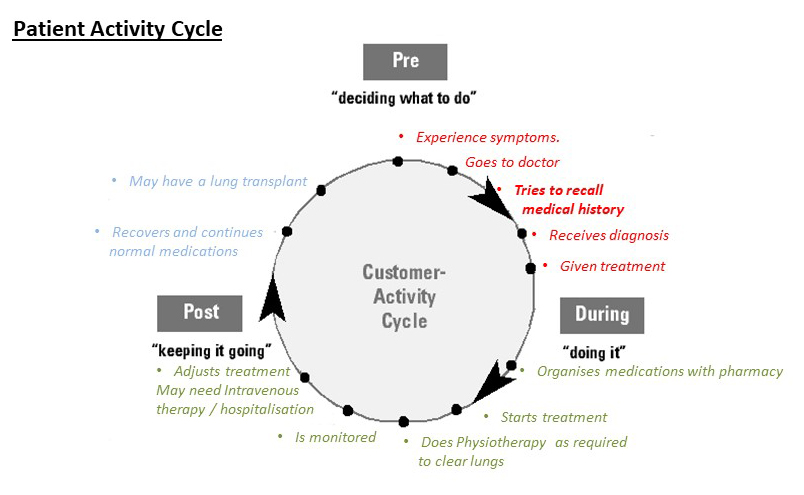

Supplement: Multimedia Appendix 4 [file jmir_v22i7e16916_app4.png]

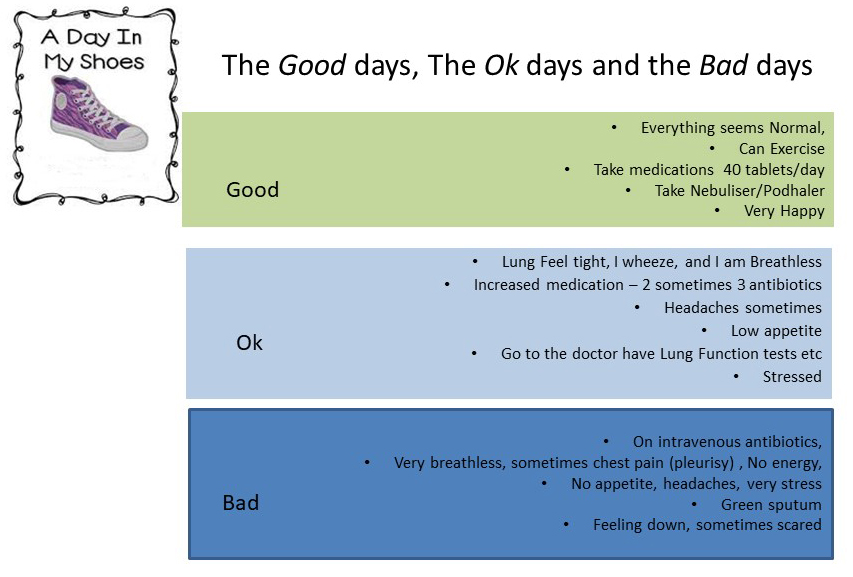

Supplement: Multimedia Appendix 5 [file jmir_v22i7e16916_app5.png]

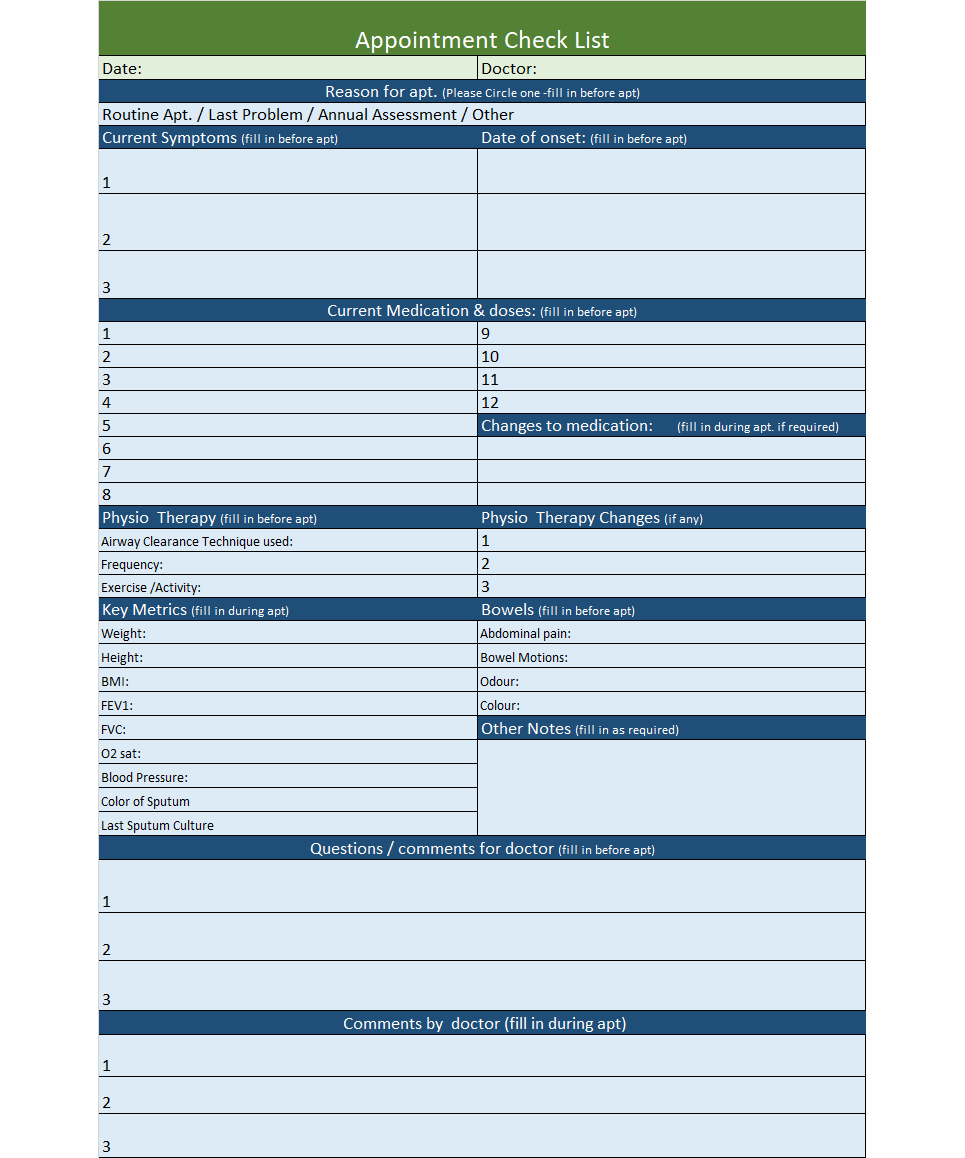

Supplement: Multimedia Appendix 6 [file jmir_v22i7e16916_app6.png]

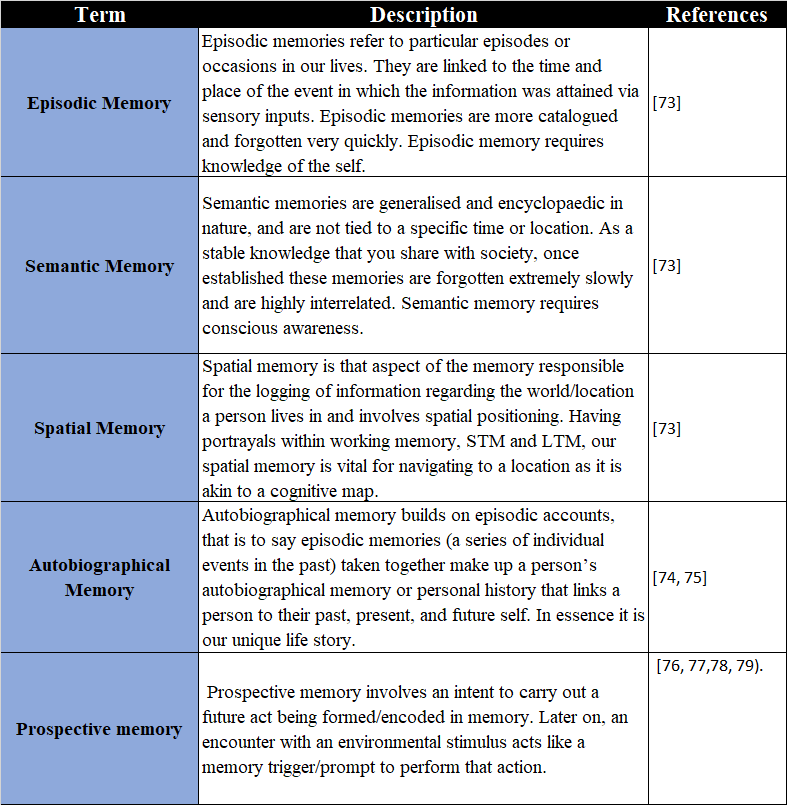

Supplement: Multimedia Appendix 7 [file jmir_v22i7e16916_app7.png]

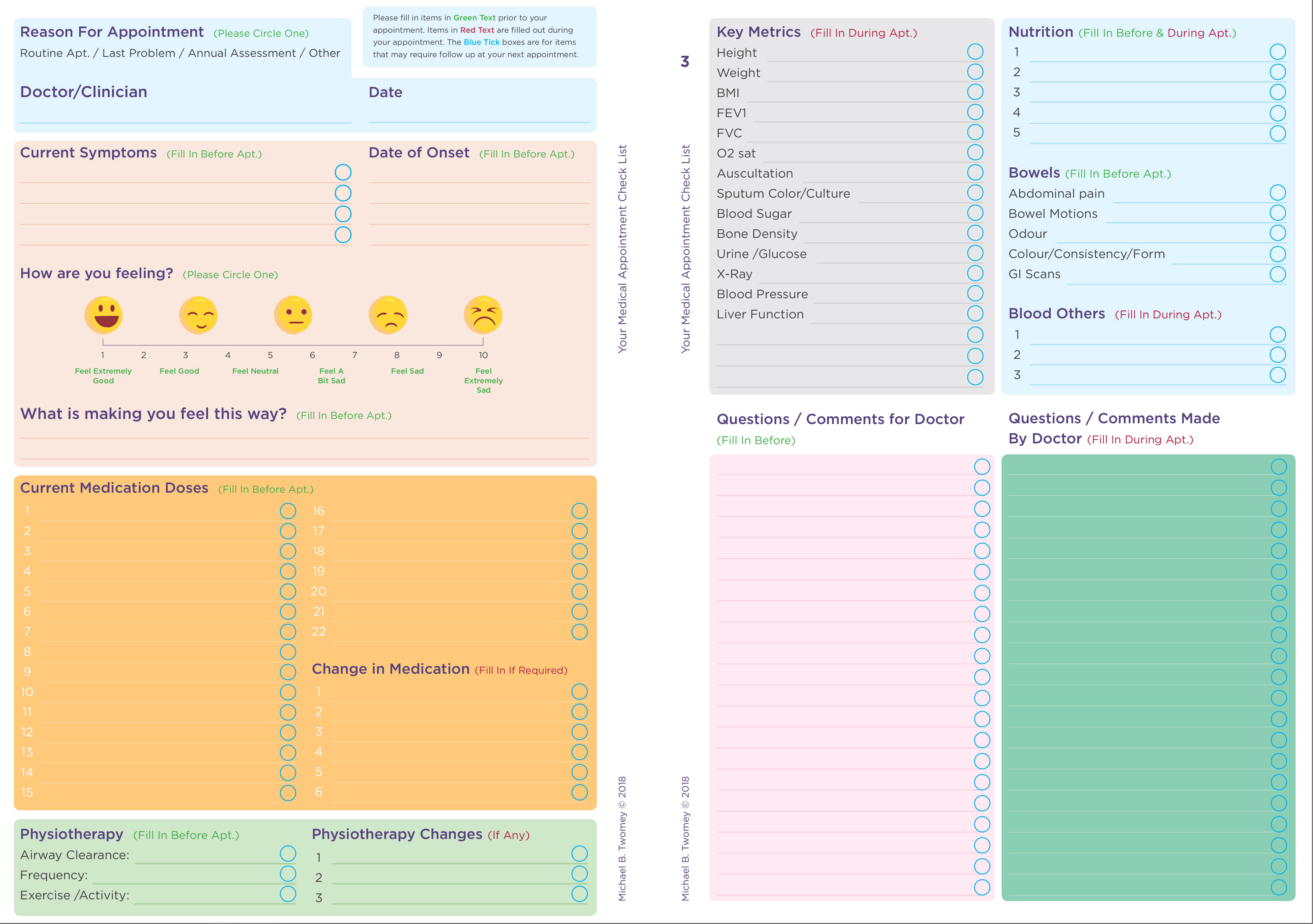

Supplement: Multimedia Appendix 8 [file jmir_v22i7e16916_app8.png]
